# Phenotypic and Genomic Characterization of *Pseudomonas wuhanensis* sp. nov., a Novel Species with Promising Features as a Potential Plant Growth-Promoting and Biocontrol Agent

**DOI:** 10.3390/microorganisms12050944

**Published:** 2024-05-07

**Authors:** Jiawei Hou, Kaiji Liao, Yong-Jie Zhang, Jun-Zhou Li, Hai-Lei Wei

**Affiliations:** 1 School of Life Science, Shanxi University, Taiyuan 030006, China; houjiawei@caas.cn (J.H.); zhangyj2008@sxu.edu.cn (Y.-J.Z.); 2 State Key Laboratory of Efficient Utilization of Arid and Semi-Arid Arable Land in Northern China, Key Laboratory of Microbial Resources Collection and Preservation, Ministry of Agriculture and Rural Affairs, Institute of Agricultural Resources and Regional Planning, Chinese Academy of Agricultural Sciences, Beijing 100081, China; liaokaiji@outlook.com

**Keywords:** *Pseudomonas wuhanensis* sp. nov., novel species, plant growth-promoting rhizobacteria, taxonomy, biological control

## Abstract

Plant growth-promoting rhizobacterial strain FP607^T^ was isolated from the rhizosphere of beets in Wuhan, China. Strain FP607^T^ exhibited significant antagonism toward several phytopathogenic bacteria, indicating that FP607^T^ may produce antimicrobial metabolites and has a stronger biocontrol efficacy against plant pathogens. Growth-promoting tests showed that FP607^T^ produced indole-3-acetic acid (IAA), NH_3_, and ferritin. The genome sequence of strain FP607^T^ was 6,590,972 bp long with 59.0% G + C content. The optimum temperature range was 25–30 °C, and the optimum pH was 7. The cells of strain FP607^T^ were Gram-negative, short, and rod-shaped, with polar flagella. The colonies on the King’s B (KB) agar plates were light yellow, smooth, and circular, with regular edges. A phylogenetic analysis of the 16S rRNA sequence and a multilocus sequence analysis (MLSA) showed that strain FP607^T^ was most closely related to the type of strain *Pseudomonas farris* SWRI79^T^. Based on a polyphasic taxonomic approach, strain FP607^T^ was identified as a novel species within the genus *Pseudomonas*, for which the name *Pseudomonas wuhanensis* sp. nov. was proposed. The type of strain used was FP607^T^ (JCM 35688, CGMCC 27743, and ACCC 62446).

## 1. Introduction

The name *Pseudomonas* was first proposed by Migula at the end of the 19th century [1]. The genus *Pseudomonas* includes a diverse microbial population with members that are derived from a wide range of environments, including water, air, soil, plants, and clinical materials [2,3,4,5,6,7,8,9,10]. According to the List of Prokaryotic Names with Standing in Nomenclature (LPSN; https://lpsn.dsmz.de (accessed on 30 December 2023)), *Pseudomonas* was the second-largest species, and more than 300 species had been validly published with correct names at the time of writing. The genus *Pseudomonas* belongs to the class *gamma-proteobacteria*, the order *Pseudomonadales*, and the family *Pseudomonadaceae*. Members of the genus *Pseudomonas* are *Gram-negative*, aerobic, non-spore-forming, and rod-shaped, with one or several polar flagella [4,5,11]. *Pseudomonas* is known for its metabolic versatility and capacity to produce abundant secondary metabolites [6,12]. They can utilize various chemical compounds as sources of carbon, nitrogen, or phosphorus, making them interesting microorganisms for processes such as bioremediation and biotransformation [2,13]. For example, *Pseudomonas mediteranea* S58 can produce siderophores, amylase, and protease, solubilize organic phosphorus, and show strong antagonism against a variety of plant pathogenic fungi and bacteria [14]. Some *Pseudomonas* spp. colonize the rhizosphere, persist throughout the growing season, and are well known for their beneficial effects on plant health. These beneficial bacteria are known as plant growth-promoting rhizobacteria (PGPR) [14,15,16]. They enhance plant growth and health through a variety of mechanisms, including accelerating germination, stimulating growth, boosting immunity, and increasing plant yields [14,16,17,18]. Furthermore, several fluorescent *Pseudomonas* exhibit diverse biocontrol mechanisms, including the production of HCN, siderophores, and antibiotics [3,16,19,20].

Given that *Pseudomonas* are potent plant growth-promoting agents, we had a comprehensive collection and identification of *Pseudomonas* strains from beet rhizosphere. In the present study, we report a novel *Pseudomonas* species isolated from beet rhizosphere in Wuhan. Strain FP607^T^ was subjected to phylogenetic analysis based on the 16S rRNA gene, multilocus sequence analysis (MLSA), average nucleotide identity (ANI), and digital DNA-DNA hybridization (dDDH) values. In vitro experiments revealed that strain FP607^T^ has a variety of characteristics that promote plant growth, including direct antagonistic activity against plant pathogens and the production of indole-3-acetic acid (IAA), NH_3_, and siderophores. A genome analysis showed that there were several genes or gene clusters in FP607^T^, which was consistent with the experimental results. This study offers a foundation for further research on how strain FP607^T^ promotes plant growth and antagonizes pathogenic microbes. In summary, they may serve as plant growth regulators and biological control agents that shield crops from harmful organisms.

## 2. Materials and Methods

### 2.1. Sample Collection and Bacterial Isolation

Samples were obtained from the rhizosphere of beets in Wuhan, China. A total of 5 g of rhizosphere soil sample was suspended in 45 mL ddH_2_O and shaken at 220 rpm for 1 h. Gradient dilution of the supernatant was performed using ddH_2_O, followed by plating on King’s B (KB) agar plates (20 g/L Bacto Tryptone, 1.5 g/L MgCl_2_, 1.5 g/L K_2_HPO_4_, and 1.5% [vol/vol] glycerol), and then incubated at 28 °C for 24 h. Individual colonies were selected using ultraviolet (UV) illumination at 254 nm and purified for three rounds. Bacterial growth was estimated through spectrophotometric measurements at optical density OD600. The cell morphology, size, and flagellar insertion were determined using the Hitachi HT7700 transmission electron microscope (TEM) at the Institute of Crop Sciences, Chinese Academy of Agricultural Sciences. Each pure culture was preserved with glycerol at a final concentration of 20% and stored in a refrigerator at −80 °C.

### 2.2. Antagonistic Test

The major host of *Clavibacter michiganense* subsp. *michiganse* (*Cmm*) is tomatoes, which causes serious economic losses [21]. As a soil-borne pathogen, *Ralstonia solanacearum* (*Ras*) naturally infects plants via its roots, causing lethal bacterial wilt disease in several types of crops [22]. Bacterial leaf streaks are an important rice disease caused by *Xanthomonas oryzae* pv. *oryzicola* spp. (*Xoc*) [23]. *X. campestris* pv. *carnpestris* (*Xcc*) can induce serious bacterial spots, causing severe damage to vegetable growth and yield [24]. *X. oryzae* pv. *oryzae* (*Xoo*) infects rice and causes bacterial leaf blight [25]. *Acidovorax citrulli* (*Ac*) causes cucurbit bacterial fruit blotch [26]. The six pathogenic bacteria were preserved in the laboratory and selected for the antagonistic test. A total of 45 mL of pre-melted nutrient agar (NA) media was incubated at 50 °C for 15 min, mixed with 5 mL of pathogenic bacterial culture, and poured onto plates. A saturated strain FP607^T^ culture (10 µL) was dropped onto the middle of the plate. The inhibitory zones were measured after 2-d of incubation at 28 °C. All the experiments were repeated over at least three rounds, and similar results were obtained.

### 2.3. Identification of Plant Growth-Promoting Traits

The typical plant-promoting traits of FP607^T^ were measured *in vitro*. IAA is a plant growth hormone secreted throughout the growth phase of plants, promotes cell proliferation, and increases plant volume and mass. It also promotes strain division and differentiation and controls other physiological functions [27]. The Salkowski colorimetric method was used to determine the ability of the bacteria to secrete the plant growth hormone IAA. Strain FP607^T^ was inoculated into a triangular flask containing KB liquid medium (containing 3 mM Tryptophan), each flask containing 20 mL of medium at 28 °C and shaken at 200 rpm for 4 d. Next, 100 μL culture solution was transferred onto a transparent plastic coagulation plate, and 100 μL Salkowski colorimetric solution (50 mL 35% HClO_4_ + 1 mL 0.5 mol/L FeCl_3_) was added. IAA (100 μL) was added to the colorimetric solution with a concentration of 100 mg/L as the positive control. Microbes produce NH_3_ by decomposing organic nitrogen molecules. A portion of the NH_3_ is absorbed by microbes or plants and converted into nitrate, which promotes plant growth. The strain FP607^T^ was transferred to peptone ammoniation medium, and the medium was mixed by shaking at 28 °C and 220 rpm for 48 h. Culture solution (200 μL) was dripped onto a white ceramic plate, and 200 μL of peptone ammoniation medium was used as a control. Three drops of Nessler’s reagent were added to the peptone ammoniation medium. Each treatment was repeated three times. The presence of yellow or brown-red precipitates indicated that the strain had NH_3_-producing capacity. Siderophore production was evaluated by overlaying chrome azurol S (CAS) medium on agar plates with the cultures [28]. Cellulase, protease, and amylase activities were determined using the clear-zone technique. Using standard techniques, the strains were evaluated for their capacity to break down potassium and organic and inorganic phosphorus.

### 2.4. Physiology and Chemotaxonomic Characterization

To identify strain FP607^T^, the physiological and biochemical characteristics were determined using Biolog GEN III MicroPlates™ (Biolog, Hayward, CA, USA). The utilization pattern was monitored on an OmniLog^®®^ Incubator/Reader (Biolog, Hayward, CA, USA). API 20NE kits (bioMérieux, Marcy l’Etoile, France) were used to determine the properties of strain FP607^T^ according to the manufacturer’s instructions. Polar lipids were detected using thin-layer chromatography (TLC). The different spots were primarily distinguished using chromogenic reagents (molybdenum phosphate, molybdenum blue, ninhydrin, D-reagent, and α-naphthol) [29]. Respiratory quinones were extracted and analyzed using high-performance liquid chromatography (HPLC; Shimadzu LC-20A) [30]. Cellular fatty acids were extracted and identified using gas chromatography (GC) (Agilent 7890 B) according to the protocol of the MIDI Sherlock Microbial Identification System and the RTSBA 6.1 database [31].

### 2.5. Molecular Identification

The amplification of a partial sequence of the 16S rRNA gene was performed using polymerase chain reaction (PCR) with the universal primers 27F (5′-GAGAGTTTGATCCTGGCTCAG-3′) and 1492R (5′-CTACGGCTACCTTGTTACGA-3′), as previously reported [32]. Strains closely related to the 16S rRNA sequence of strain FP0607^T^ were identified using the EzBioCloud database [33]. The PCR products were sequenced by GENEWIZ Ltd. (Suzhou, China). The housekeeping genes *gyrB*, *rpoB*, *rpoD*, and 16s rRNA were obtained from the MLSA database (http://microbiologia.uib.es/bioinformatica/ (accessed on 31 December 2023)) using publicly available genomic sequences [34]. Phylogenetic trees were constructed using the neighbor-joining (NJ) method in MEGA 11 [35]. Bootstrap values were calculated for 1000 replications. Kimura’s two parameters were selected as the nucleotide substitution model to compute the evolutionary distances, and the bootstrap values were derived from 1000 bootstrap replications [36]. The ANI values between FP607^T^ and closely related strains’ orthologous average nucleotide identity (OrthoANI) were calculated using an online EzBioCloud server (https://www.ezbiocloud.net/tools/ani (accessed on 30 November 2023)) [37]. The pairwise dDDH values were investigated by comparing genome sequences using the Genome–Genome Distance Calculator (GGDC, http://ggdc.dsmz.de/distcalc2.php (accessed on 30 November 2023)), using the alignment method Basic Local Alignment Search Tool (BLAST+) and Formula 2 for incomplete genome sequences [38].

### 2.6. Genome Sequencing, Annotation, and Comparative Genomic Analysis

The genomic DNA of FP607^T^ was extracted using a commercial genomic DNA extraction kit (Omega Bio-Tek, Norcross, GA, USA). Whole-genome sequencing (WGS) was performed by GENEWIZ Ltd., (Suzhou, China). Coding sequences (CDSs) were predicted using the Prodigal software and annotated against the National Center for Biotechnology Information (NCBI)’s non-redundant (NR) database. RNA sequences were predicted using the tRNAscan-SE and RNAmmer software (https://services.healthtech.dtu.dk/services/RNAmmer-1.2/ (accessed on 30 November 2023)) [39]. Functional annotation was performed using the Cluster of Orthologous Groups (COG) and Kyoto Encyclopedia of Genes and Genomes (KEGG) databases [14]. Potential secondary metabolic gene clusters were predicted using antiSMASH 7.0 [40]. CRISPR-Cas sequences were predicted using CRISPRCasFinder (https://crisprcas.i2bc.paris-saclay.fr/CrisprCasFinder/Index (accessed on 30 November 2023)) [41]. MacSyFinder 2.1.2 was used to identify the secretion systems [42]. Genes in the genome can produce phenotypes that benefit plant growth. BLASTP was used to search for homologs with a threshold of 1e^−5^ [43]. Marker genes, including genes related to 1-ami-nocyclopropane-1-carboxylic acid (ACC) deaminase, indole-3-acetyl-aspartic acid hydrolase (IAA-Asp), β-galactosidase, amylase, pectinase, cellulase, and protease, were obtained from the UniProt database [44].

### 2.7. Statistical Analysis

All the experiments were repeated in triplicate, and the means and SD are shown in this article. Tukey’s Honestly Significant Difference (HSD) test (*p* < 0.05) was used for the statistical analysis of the results.

## 3. Results and Discussion

### 3.1. Antagonism against Phytopathogens

**Figure 1:**
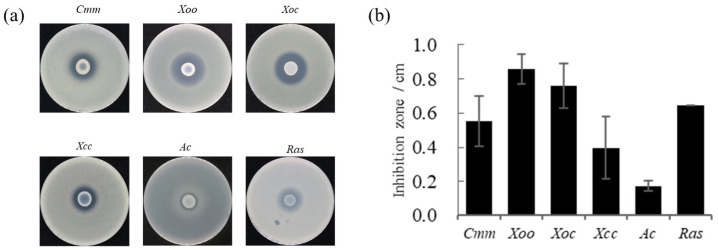
In vitro antimicrobial activity of strain FP607^T^ against various phytopathogens. (**a**) Six plant bacterial pathogens have been selected to test the antimicrobial activity of strain FP607^T^. (*Cmm*, *Clavibacter michiganense* subsp. *michiganse. Xoo*, *Xanthomonas oryae* pv. *oryzae* PXO99. *Xoc*, *Xanthomonas oryzae* pv. *oryzicola* RS105. *Xcc*, *Xanthomonas campestris* pv. *carnpestris. Ac*, *Acidovorax avenae. Ras,* and *Ralstonia solanacearum*). (**b**) Antimicrobial activity has been estimated by measuring the diameter (mm) of the clear zone of growth inhibition from Figure 1a.

Strain FP607^T^ had an antagonistic effect on pathogenic bacteria. The pathogenic bacteria tested included *Clavibacter michiganense* subsp. *michiganse* (*Cmm*), *Xanthomonas oryae* pv. *oryzae* PXO99 (*Xoo*), *X. oryzae* pv. *oryzicola* RS105 (*Xoc*) and *X. campestris* pv. *carnpestris* (*Xcc*), *Ralstonia solanacearum* (*Ras*), and *Acidovorax avenae* (*Ac*). A prominent bacteriostatic circle was observed on the plates, indicating that strain FP607^T^ can inhibit the growth of *Cmm, Xoo*, *Xoc*, *Xcc*, *Ac*, and *Ras* (Figure 1). This further implies that FP607^T^ can inhibit pathogenic bacteria through metabolite production rather than through direct contact.

### 3.2. Plant Growth-Promoting Activities

**Figure 2:**
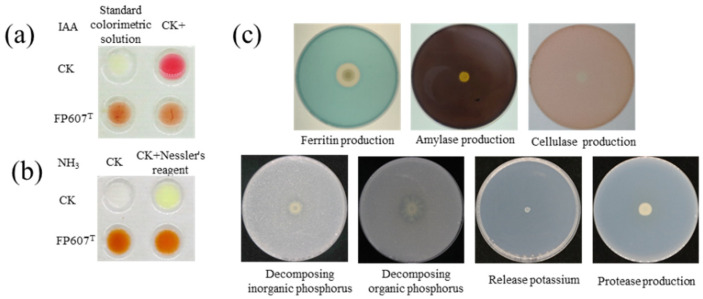
In vitro test of plant growth-promoting traits of strain FP607^T^. (**a**) Indole-3-acetic acid (IAA) production is shown by the visualized pink color treated with strain FP607^T^ compared with the upper positive and negative controls. CK+ indicates that 100 μL/L of IAA was added to the colorimetric solution. (**b**) Ammonia production is shown from the visualized brown and yellow colors treated with FP607^T^ (lower two replicates) compared with the water control. (**c**) Response of strain FP607^T^ in various identification media. The transparent zones around the colonies are visualized through siderophore production.

The Salkowski colorimetric results showed that strain FP607^T^ could produce IAA because it caused the colorimetric solution to turn red upon introduction (Figure 2a). The culture broth and peptone-ammoniated medium produced a brownish-colored precipitate when three drops of Nath’s reagent were added, suggesting that the strain could produce NH_3_ (Figure 2b). Selective media were used to characterize several features that promoted plant development. Strain FP607^T^ developed siderophores, as shown by the distinct hydrolytic halos that formed around the colonies. However, strain FP607^T^ did not show a translucent aperture on subsequent identification plates, indicating that it was unable to produce cellulase, amylase, or proteases or break down potassium phosphorus, inorganic phosphorus, or organic phosphorus (Figure 2c). In conclusion, we speculated that FP607^T^ may promote plant growth.

### 3.3. 16S rRNA and MLSA Phylogenies

**Figure 3:**
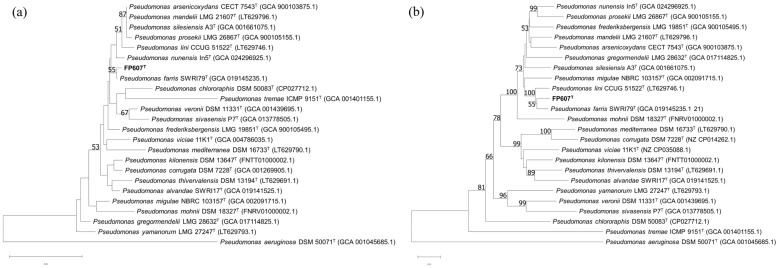
(**a**) NJ tree based on the nearly complete 16S rRNA gene sequences showing the relationships between FP607^T^ and other type strains. (**b**) NJ tree based on concatenated sequences of 16S rRNA, *gyrB*, *rpoB*, and *rpoD* genes showing the phylogenetic relationship between FP607^T^ and other type strains. Bootstrap values > 50% (based on 1000 resamplings) are shown. Scale bar: 0.01 substitutions per nucleotide position.

To more accurately identify the bacterium, we performed a phylogenetic analysis of the 16S rRNA gene. The phylogenetic tree based on the 16S rRNA gene’s sequences constructed using the NJ method indicated that FP607^T^ formed a cluster along with *Pseudomonas farris* SWRI79^T^, with a bootstrap value of 55%. (Figure 3a) [45]. Concatenated sequences of the 16S rRNA, *gyrB*, *rpoB*, and *rpoD* genes were used to reconstruct a phylogenetic tree, using the same approach. The MLSA tree showed that FP607^T^ and *Pseudomonas farris* SWRI79^T^ were most closely related, with a bootstrap value of 55% (Figure 3b).

The results of the whole-genome similarity test showed that the values between FP607^T^ and other closely related species ranged between 86.7 and 93.6% for ANI and between 31.1 and 66.5% for dDDH, below the recommended thresholds of 95% (ANI) and 70% (dDDH) for prokaryotic species delineation, respectively (Table 1). This indicated that strain FP607^T^ may be a new species. Strain FP607^T^ was named *P. wuhanensis* FP607^T^ and was deposited in the China General Microorganism Culture Collection (CGMCC 27743), the Japan Collection of Microorganisms (JCM 35688), and the Agriculture Culture Collection of China (ACCC 62446).

### 3.4. Physiology and Chemotaxonomic Characterization

**Figure 4:**
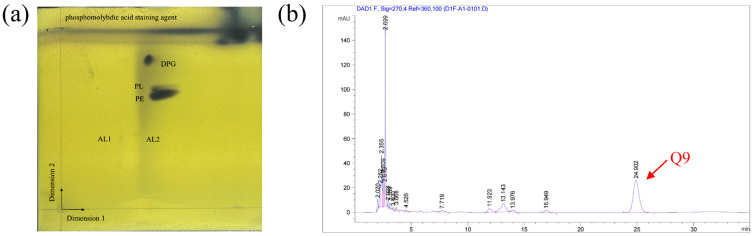
(**a**) Two-dimensional (2D) TLC plate of polar lipids extracted from strain FP607^T^. Three polar lipids (DPG, PL, and PE) were detected. PE, phosphatidylethanolamine; DPG, diphosphatidylglycerol; PL, unidentified phosphoglycolipid; APL, aminophospholipid; and L, unknown polar lipids. (**b**) The quinone species Q-9 was detected after 25 min during the HPLC analysis.

The Biolog GEN III MicroPlate™ system was used to test the carbon source utilization and chemical sensitivity of FP607^T^. Strain FP607^T^ could use D-mannose, D-galactose, D-fucose, guanidine HCl, L-galactonic acid lactone, D-glucuronic acid, glucuronamide, and α-D-glucose as carbon sources for growth (Supplementary Materials Table S2), and it exhibited chemical sensitivity to sodium lactate, troleandomycin, rifamycin SV, and lincomycin. The API 20NE results showed that strain FP607^T^ reacted with potassium nitrate, N-acetyl glucosamine, and citric acid (Supplementary Materials Table S2). The analysis of the polar lipids revealed that the cell membrane of strain FP607^T^ contained diphosphatidylglycerol (DPG), an unidentified phosphoglycolipid (PL), and phosphatidylethanolamine (PE) (Figure 4a). The primary respiratory quinone of strain FP607^T^ was ubiquinone-9 (Q-9), as shown by the peak formed in 25 min during the HPLC analysis (Figure 4b). The major fatty acids of strain FP607^T^ contained C_12:0_ (13.9%), C_10:0_ 3-OH (39.0%), C_12:0_ 2-OH (11.4%), C_12:0_ 3-OH (18.1%), and summed features 3 (C_16:1_ ω7c/C_16:1_ ω6c) (7.1%) and 8 (C_18:1_ ω7c/C_18:1_ ω6c) (Table 2). These findings are consistent with previous reports on the *Pseudomonas* species [26].

### 3.5. Genomic Characterization

FP607^T^ had a genome of 6,590,972 bp with a G + C 58.99% content, and it was predicted to contain 6011 coding sequences (CDSs) with an average length of 992.90 bp. In addition, *P. wuhanensis* FP607^T^ encoded 19 rRNA genes, 70 tRNAs, and 209 ncRNAs (Supplementary Materials Table S1 and Figure S1).

FP607^T^ was equipped with several secretion systems, including type I secretion system (T1SS), type II secretion system (T2SS), type III secretion system (T3SS), and type VI secretion system (T6SS) (Supplementary Materials Figure S2), out of which T3SS and T6SS have been demonstrated to be involved in regulating plant immunity and promoting bacterial colonization and plant growth [46]. For instance, *Pseudomonas fluorescens* F113 employed T6SS to mediate bacterial killing and adaption to rhizosphere colonization [46]. Whether these secretion systems contribute to the antagonistic activity and benefic function of FP607^T^ remains to be investigated. The in silico prediction revealed that almost all the closely related strains contained plant growth-promotion-associated genes encoding 1-aminocyclopropane-1-carboxylic acid (ACC) deaminase and indoleacetamide hydrolase (iaaH). Moreover, strain FP607^T^ showed genes encoding digestive enzymes, such as dextranase amylase and protease, but did not contain the genes for β-galactosidase, pectinase, or cellulase (Supplementary Materials Figure S2). The antiSMASH results identified 18 potential secondary metabolite biosynthesis gene clusters, including redox-cofactor, NRPS, NRP-metallophore, HR-T2PKS, isocyanide, RiPP-like, beta-lactone, NRPS-like, and aryl polyene (Table 3). Of these, 11 were similar to known biosynthetic gene clusters (BGCs), including Lankacidin, Pf-5, pyoverdine, histicorrugatin, cepacin A, pyoverdine DC3000, bacillomycin D, fengycin, pyralomicin 1a, APE Vf, and fragments. Histicorrugatin is a siderophore structurally related to corrugatin and ornicorrugatin. They can also mediate the synthesis of iron carriers. The mechanisms of action of bacterial siderophores against plant pathogenic fungi include nutrient competition, niche competition, the induction of systemic resistance in plants, and the disruption of pathogen iron homeostasis [47]. Pyoverdine (Pvd) is a major iron-chelating metabolite of *Pseudomonas* proteins, and its functions include antibiotic activity and metal homeostasis [47,48]. In conclusion, we speculate that strain FP607^T^ showed a growth-promoting effect possibly because it contained secondary metabolites of siderophores. Lankacidin, cepacin A, bacillomycin D, fengycin, and its fragments are well-known antagonists. Lankacidins are a class of natural polyketide products that exhibit promising antimicrobial activity [49]. Cepacin A is an important metabolite that protects sprouted peas from moisture damage caused by *Globisporangium ultimum* [50]. Bacitracin D-C16 is a naturally occurring antimicrobial lipopeptide that inhibits the growth of *Fusarium verticillioides* in maize [51]. The fengycin family is one of the most important molecules that affect target cells at the membrane level and has a broad spectrum of antagonistic activity against plant pathogens [52]. Fragin is a metallophore, and metal chelation is the molecular basis of its antifungal activity [53]. According to this analysis, the strain FP607^T^ possesses genes that result in the production of the aforementioned secondary metabolites, which may be crucial for the antagonistic action of the strain against harmful bacteria.

The COG annotation results indicated that the largest COG categories were general function prediction only (COG R), amino acid transport and metabolism (COG E), and unknown function (COG S) (Supplementary Materials Figure S3). The KEGG annotation results revealed that carbohydrate metabolism (444 genes), amino acid metabolism (435 genes), and global and overview maps (375 genes) were the most abundant categories in FP607^T^. Several genes were involved in membrane transport (279 genes) and signal transduction (242 genes) (Supplementary Materials Figure S4).

### 3.6. Description of P. wuhanensis sp. nov.

The type strain *P. wuhanensis* FP607^T^ was isolated from the rhizosphere of beets in Wuhan, China. The genome of *P. wuhanensis* FP607^T^ was 6,590,972 bp with 59.0% GC content. The cells of strain FP607^T^ were Gram-negative and rod-shaped (length 2.0–2.7 µm, width 0.9–1.2 µm), with polar flagella (Supplementary Materials Figure S5). The colonies on KB were light yellow, circular, and smooth with regular edges. The optimum temperature range was 25–30 °C, and the optimum pH was 7. *P. wuhanensis* FP607^T^ could produce IAA, NH_3_, and ferritin and had plant growth-promoting functions. *P. wuhanensis* FP607^T^ significantly inhibited *Cmm, Xoo*, *Xoc*, *Xcc*, *Ac*, and *Ras*. The API 20NE results showed that the strain *P. wuhanensis* FP607^T^ could react with potassium nitrate, N-acetyl glucosamine, and citric acid. The analysis of polar lipids revealed that the cell membrane of strain *P. wuhanensis* FP607^T^ contained diphosphatidylglycerol (DPG), an unidentified phosphoglycolipid (PL), and phosphatidylethanolamine (PE). The BIOLOG results showed that strain *P. wuhanensis* FP607^T^ could utilize D-mannose, D-galactose, D-fucose, guanidine HCl, L-galactonic acid lactone, D-glucuronic acid, glucuronamide, and α-D-glucose as carbon sources for growth (Supplementary Materials Table S2) and exhibited chemical sensitivity to sodium lactate, troleandomycin, rifamycin SV, and lincomycin. The primary fatty acids of strain *P. wuhanensis* FP607^T^ were C_12:0_, C_10:0_ 3-OH, C_12:0_ 2-OH, C_12:0_ 3-OH, and summed features 3 (C_16:1_ ω7c/C_16:1_ ω6c), and 8 (C_18:1_ ω7c/C_18:1_ ω6c). The predominant isoprenoid quinone was ubiquinone-9. The phylogenetic analysis showed that FP607^T^ was closely related to *Pseudomonas farris* SWRI79^T^. The ANI and dDDH values showed that strain FP607^T^ was closely related to *Pseudomonas farris* SWRI79^T^, with values of 93.6% and 66.5%, respectively. Collectively, the type strain *P. wuhanensis* FP607^T^ (=JCM 35,688 = CGMCC 27,743 = ACCC 62,446) was identified in this study.

## 4. Conclusions

*Pseudomonas,* as the second-largest species, are valuable microbial resources used widely in agriculture and industry [54,55,56,57,58]. This study focused on a newly discovered, patented strain of *P. wuhanensis* FP607^T^ with plant-promoting properties. Strain FP607^T^ is rich in secondary metabolites of potential growth-promoting gene clusters and can produce IAA, NH_3_, and siderophores, suggesting that strain FP607^T^ is a potent PGPR with versatile beneficial characteristics. The differences in the phenotypic characteristics between the novel isolates and their phylogenetic neighbors are summarized in this paper. Morphological, chemotaxonomic, and phylogenetic analyses strongly supported the affiliation of strain FP607^T^ as a novel species within *Pseudomonas*. Several characteristic features, such as physiological and biochemical characteristics, 16S rRNA gene sequences, and the MLSA, ANI, and DDH values, can be used to distinguish this strain from phylogenetically related taxa. *P. wuhanensis* FP607^T^ is a plant rhizosphere probiotic that is easy to cultivate and preserve, highly efficient to use, and environmentally friendly. Collectively, the discovery of this new *Pseudomonas* strain is of great significance for agricultural production and supports the expansion of new resources.

## Figures and Tables

**Table 1:** The dDDH and ANI values between strain FP607^T^ and the type strains of closely related species of the genus *Pseudomonas.*

Species	dDDH (%)	ANI (%)
	FP607^T^	FP607^T^
*Pseudomonas farris* SWRI79^T^	66.5	93.6
*Pseudomonas lini* DSM 16768^T^	54.2	94.0
*Pseudomonas frederiksbergensis* LMG 19851^T^	35.9	88.8
*Pseudomonas silesiensis* A3^T^	34.1	87.5
*Pseudomonas mandelii* LMG 21607^T^	34.1	88.1
*Pseudomonas arsenicoxydans* CECT 7543^T^	33.5	87.9
*Pseudomonas moorei* DSM 12647^T^	31.6	86.8
*Pseudomonas mohnii* DSM 18327^T^	31.5	86.7
*Pseudomonas jessenii* LMG 21605^T^	31.2	86.9
*Pseudomonas laurylsulfatiphila* AP3 16^T^	31.1	86.7

**Table 2:** Cellular fatty acid composition of strain FP607^T^ and reference strains.

Fatty Acid	1	2	3	4	5
Summed features: *					
3(C_16:1_ ω7c/C_16:1_ ω6c)	7.1	28.0	31.2	34	36.9
8(C_18:1_ ω7c/C_18:1_ ω6c)	1.5	8.4	4.8	11.9	18.3
Saturated:					
C_12:0_	13.9	10.3	4.1	2.1	6.5
C_16:0_	1.7	16.1	19.5	32.1	27.9
Hydroxy:					
C_10:0_ 3-OH	39.0	7.6	8.8	3.8	3.7
C_12:0_ 2-OH	11.4	5.4	8.9	4.1	1.0
C_12:0_ 3-OH	18.1	6.7	7.4	4.3	3.1

Strains: 1, *P. wuhanensis* FP607^T^; 2, *P. lini* CCUG 51522^T^; 3, *P. frederiksbergensis* DSM 13022^T^; 4, *P. arsenicoxydans* DSM 27171^T^; and 5, *P. silesiensis* A3^T^. * Summed features represent groups of two fatty acids that could not be separated using the Mid-Sherlock system.

**Table 3:** Putative BGCs in the FP607^T^ genome predicted by antiSMASH.

Region	Type	Most Similar Known Cluster	Location	BGC Type
1	redox-cofactor	Lankacidin	603,022–625,194	NRP + Polyketide
2	Ripp-like	-	727,901–740,105	-
3	lanthipeptide class II	-	880,816–903,950	-
4	Ripp-like	-	982,561–994,438	-
5	NAGGN	-	1,814,162–1,829,030	-
6	NRPS	Pf-5 pyoverdine	1,969,442–2,022,425	NRP
7	NRP-metallophore	histicorrugatin	2,829,428–2,905,150	NRP
8	HR-T2PKS	cepacin A	2,933,224–2,975,466	Polyketide
9	hydrogen-cyanide	-	3,343,615–3,356,462	-
10	isocyanide	pyoverdine DC3000	3,844,839–3,886,506	NRP
11	Ripp-like	bacillomycin D	3,926,086–3,936,925	Polyketide + NRP: Lipopeptide
12	betalactone	fengycin	4,157,666–4,180,849	NRP
13	hydrogen-cyanide	-	4,181,489–4,194,536	-
14	NRPS-like	pyralomicin 1a	4,454,343–4,509,652	NRP + Polyketide: Modular type I polyketide
15	NRP-metallophore	Pf-5 pyoverdine	4,547,832–4,633,873	NRP
16	Ripp-like	-	5,225,729–5,236,583	-
17	Aryl polyene	APE Vf	6,109,423–6,152,929	Other
18	NRPS-like	fragin	6,413,864–6,457,280	NRP

## Data Availability

The datasets supporting the conclusion of this article are included in the article and Supplementary Materials. Further inquiries can be directed to the corresponding authors.

## Supplementary Materials

The following supporting information can be downloaded at: https://www.mdpi.com/article/10.3390/microorganisms12050944/s1, Figure S1: The genome circle map of strain FP607^T^; Figure S2: Selected biosynthetic, secretion system-related, and catabolic genes or gene clusters were predicted using the genome sequences of strain *P. wuhanensis* FP607^T^ and the related species; Figure S3: COG annotation; Figure S4: KEGG annotation; Figure S5: Micrograph of TEM of cells of strain FP607^T^; Table S1: General features of *P. wuhanensis* FP607^T^ genome; and Table S2: Phenotypic characters that differentiate FP607^T^ from its closest type strains.
