# Supplementary material for: Phenotypic and Genomic Characterization of Pseudomonas wuhanensis sp. nov., a Novel Species with Promising Features as a Potential Plant Growth-Promoting and Biocontrol Agent
Source: Microorganisms. 2024 May 7;12(5):944. doi: 10.3390/microorganisms12050944 (PMC11124405; doi:10.3390/microorganisms12050944)
Supplement: Supplementary file 1 [file microorganisms-12-00944-s001.zip › microorganisms-2951623-supplementary.pdf]

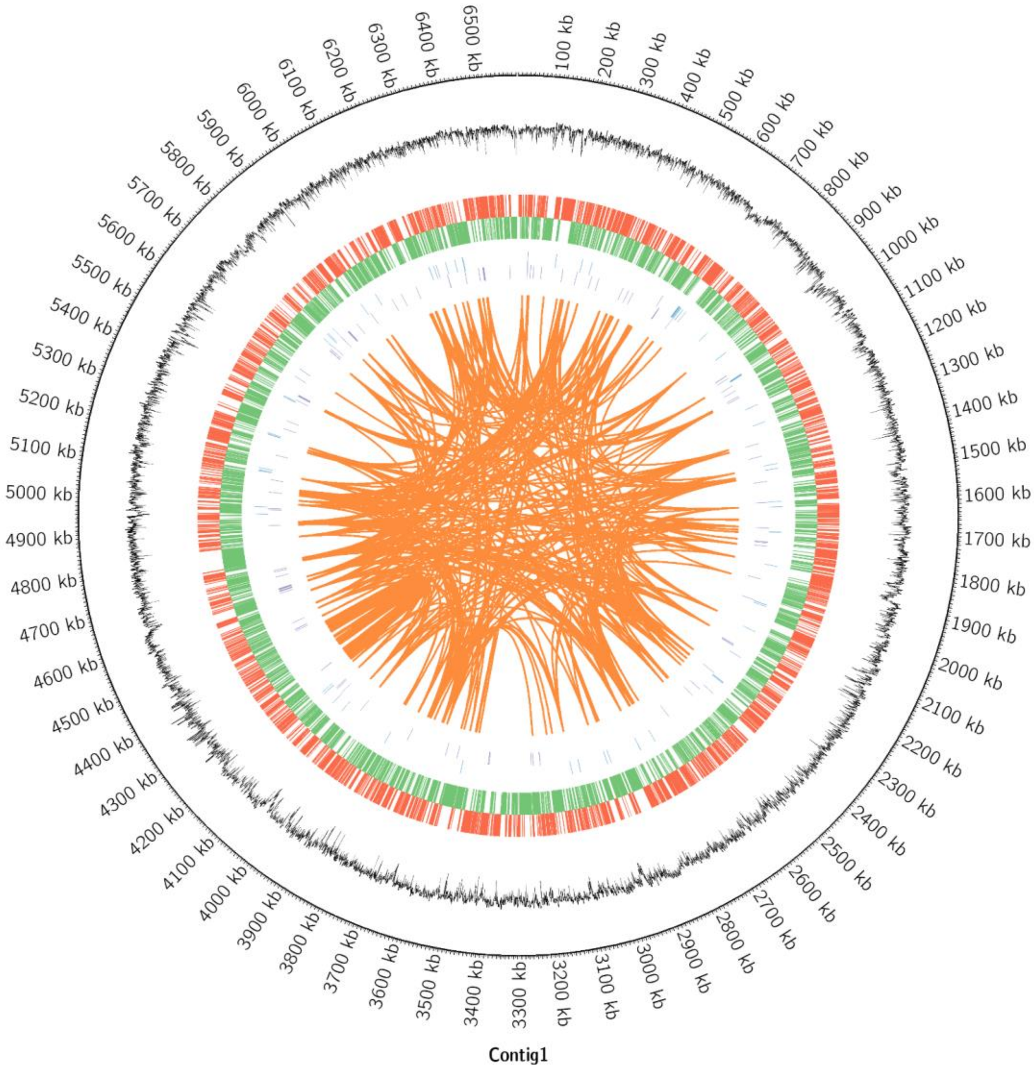

Figure S1. The genome circle map of strain FP607^T.
Note: The circles from outside to inside are: The first circle is genomic location information, the second circle is GC content information, the third circle is the coding genes on the positive chain (marked in red), the fourth circle is the coding genes on the negative chain (marked in green), the fifth circle is the ncRNA information on the positive chain (marked in blue), the sixth circle is the ncRNA information on the negative chain (marked in purple), and the seventh circle is the long fragment of repetitive sequence information in the genome (marked in orange).

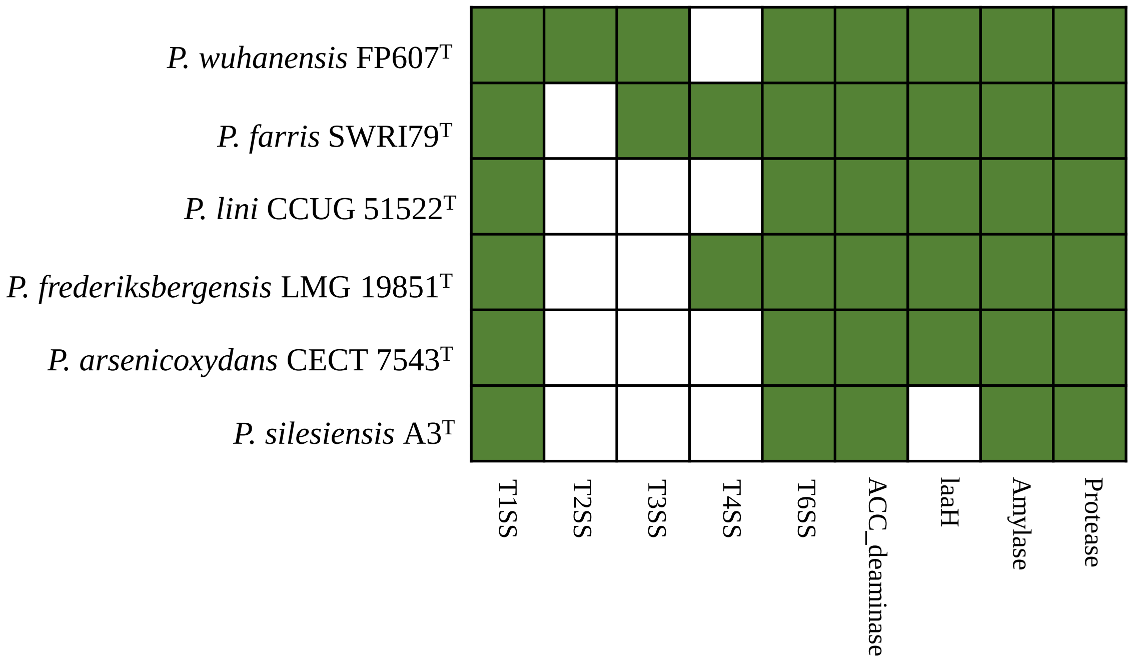

Figure S2 . Selected biosynthetic, secretion system-related and catabolic genes or gene clusters were predicted using the genome sequences of strain *P. wuhanensis* FP607^{T} and the related species.

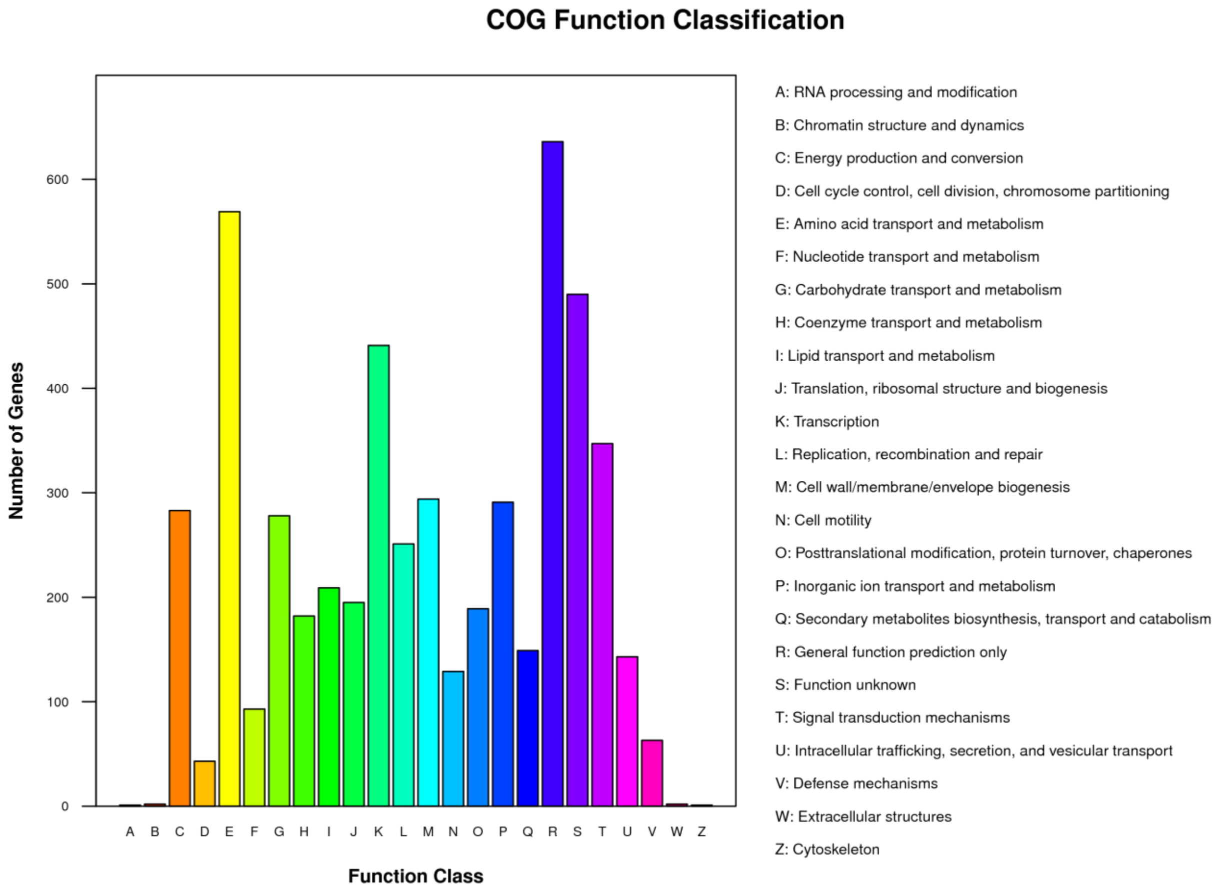

Figure S3. COG annotation. Different colors indicate different functional categories.

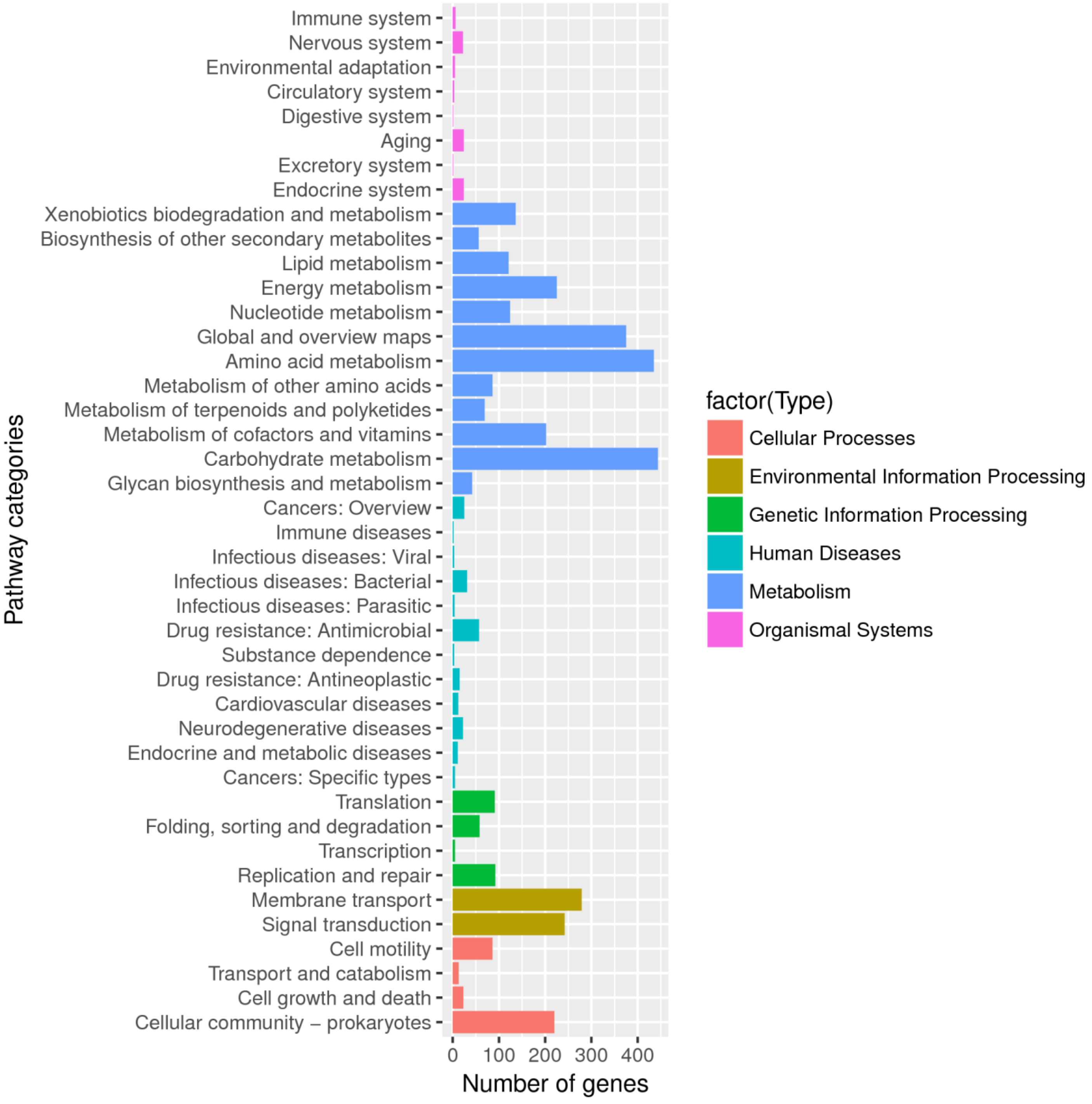

Figure S4. KEGG annotation. Different colors indicate different pathway categories.

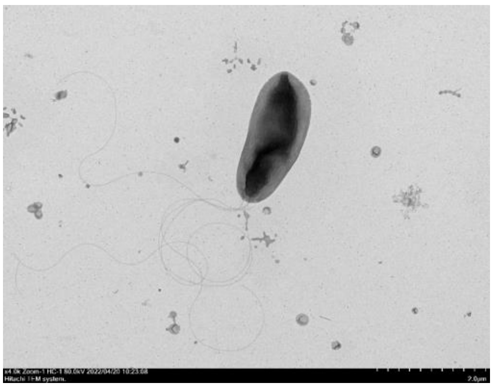

Figure S5. Micrograph of TEM of cells of strain FP607^{T}. Strain FP607^{T} had an average size of 2.5 × 1.1 μm (length 2.0–2.7 μm, width 0.9–1.2 μm).

Table S1. General features of the *P. wuhanensis* FP607^T genome.

**Features**	**Chromosome**
**Size (bp)**	6,590,972
**G+C content (%)**	58.99
**Number of total CDSs**	6011
**tRNAs**	70
**rRNA genes**	19
**ncRNAs**	209
**Contigs**	1
**Total CDSs size (bp)**	5,773,998
**Average CDS length (nt)**	960.57

Table S2. Phenotypic characters that differentiate FP607^T from its closest type strains.

**Characteristic**	1	2	3	4	5
Temperature range for growth (°C)	4-36℃	4-36℃	4-30℃	4-37℃	4-35℃
NaCl range for growth (%, w/v)	0-3%	0-3%	0-4%	0-2%	0-4%
pH range for growth	6-9	6-8	4-9	6.5-10	6-9
**API 20 NE tests:**					
Potassium Nitrate	+	+	+	-	+
L-Arginine	-	-	-	+	-
N-acetyl glucosamine	+	+	+	+	-
D-Maltose	-	-	-	+	+
Citric acid	+	+	+	+	-
**Carbon source utilization assays (Biolog GENIII):**					
L-Fucose	-	w	-	+	+
Inosine	-	-	-	+	+
1% Sodium Lactate	+	w	-	+	+
Fusidic Acid	-	+	-	+	+
D-Serine	-	+	-	+	+
α-D-Glucose	+	+	-	+	+
D-Mannose	+	+	-	+	+
D-Fructose	w	+	-	+	+
D-Galactose	+	+	-	+	+
D-Fucose	+	+	-	+	+
D-Sorbitol	-	-	-	+	+
D-Mannitol	-	-	-	+	+
D-Arabitol	-	-	-	+	+
Glycerol	-	-	-	+	+
D-Serine	-	-	-	+	+
Glycyl-L-Proline	-	-	-	+	+
L-Alanine	-	-	-	+	+
L-Aspartic Acid	-	-	-	+	+
L-Histidine	-	-	-	+	+
L-Pyroglutamic Acid	-	-	-	+	+
L-Galactonic Acid Lactone	+	-	-	+	+
Mucic Acid	+	-	-	+	+
Vancomycin	+	w	w	+	+
Methyl Pyruvate	-	-	-	+	+
L-Lactic Acid	-	-	-	+	+
Troleandomycin	+	+	-	+	+
Lincomycin	+	+	-	+	+
α-Keto-Glutaric Acid	-	-	-	+	+
D-Malic Acid	-	-	-	+	+
L-Malic Acid	-	-	-	+	+
Bromo-Succinic Acid	-	-	-	+	+
Nalidixic Acid	-	-	-	+	+
Tween 40	-	-	w	+	+
α-Hydroxy-Butyric Acid	-	-	-	+	+
β-Hydroxy-D,L-butyric Acid	-	-	-	+	+
α-Keto-Butyric Acid	-	-	-	+	+
Acetoacetic Acid	-	-	-	+	+
Propionic Acid	-	-	-	+	+
Formic Acid	-	-	-	+	+
Aztreonam	+	-	-	+	+
Sodium Butyrate	+	-	-	+	-
Sodium Bromate	-	-	-	+	+

*Strains: 1, ***P. wuhanensis*** **FP607^T**; 2, *P. lini* CCUG 51522^T; 3, *P. frederiksbergensis* DSM 13022^T; 4, *P. arsenicoxydans* DSM 27171^T; 5, *P. silesiensis* A3^T.

* +, positive; −, negative; w, weak.
